# *PAN3*–*PSMA2* fusion resulting from a novel t(7;13)(p14;q12) chromosome translocation in a myelodysplastic syndrome that evolved into acute myeloid leukemia

**DOI:** 10.1186/s40164-018-0099-4

**Published:** 2018-03-20

**Authors:** Ioannis Panagopoulos, Ludmila Gorunova, Hege Kilen Andersen, Astrid Bergrem, Anders Dahm, Kristin Andersen, Francesca Micci, Sverre Heim

**Affiliations:** 1 Section for Cancer Cytogenetics, Institute for Cancer Genetics and Informatics, The Norwegian Radium Hospital, Oslo University Hospital, Montebello, PO Box 49534 Nydalen, 0424 Oslo, Norway; 2 Department of Haematology, Akershus University Hospital, Nordbyhagen, Norway; 3 Faculty of Medicine, Institute of Clinical Medicine, University of Oslo, Oslo, Norway

**Keywords:** Myelodysplastic syndrome, Acute myeloid leukemia, Chromosome translocation, t(7;13)(p14;q12), RNA sequencing, *PAN3*–*PSMA2* fusion, PAN2/PAN3 complex, Proteasome

## Abstract

**Background:**

Acquired primary chromosomal changes in cancer are sometimes found as sole karyotypic abnormalities. They are specifically associated with particular types of neoplasia, essential in establishing the neoplasm, and they often lead to the generation of chimeric genes of pathogenetic, diagnostic, and prognostic importance. Thus, the report of new primary cancer-specific chromosomal aberrations is not only of scientific but also potentially of clinical interest, as is the detection of their gene-level consequences.

**Case presentation:**

RNA-sequencing was performed on a bone marrow sample from a patient with myelodysplastic syndrome (MDS). The karyotype was 46,XX,t(7;13)(p14;q12)[2]/46,XX[23]. The MDS later evolved into acute myeloid leukemia (AML) at which point the bone marrow cells also contained additional, secondary aberrations. The 7;13-translocation resulted in fusion of the gene *PAN3* from 13q12 with *PSMA2* from 7p14 to generate an out-of-frame *PAN3*–*PSMA2* fusion transcript whose presence was verified by RT-PCR together with Sanger sequencing. Interphase fluorescence in situ hybridization analysis confirmed the existence of the chimeric gene.

**Conclusions:**

The novel t(7;13)(p14;q12)/*PAN3*–*PSMA2* in the neoplastic bone marrow cells could affect two key protein complex: (a) the PAN2/PAN3 complex (*PAN3* rearrangement) which is responsible for deadenylation, the process of removing the poly(A) tail from RNA, and (b) the proteasome (*PSMA2* rearrangement) which is responsible for degradation of intracellular proteins. The patient showed a favorable response to decitabine after treatment with 5-azacitidine and conventional intensive chemotherapy had failed. Whether this might represent a consistent feature of MDS/AML with this particular gene fusion, remains unknown.

## Background

Hematologic malignancies, including acute myeloid leukemia (AML) and acute lymphoblastic leukemia (ALL), often carry visible acquired chromosomal aberrations which may be either primary or secondary in leukemogenesis [1]. According to Heim and Mitelman [1] “Primary aberrations are frequently found as the sole karyotypic abnormalities in cancer and are often specifically associated with particular tumor types. The term primary not only refers to the fact that these are the first changes we see in neoplastic cells, but also reflects their causal role in tumorigenesis; they are essential in establishing the neoplasm”. In the same reference secondary aberrations are described as “rarely or never found alone; as the name implies, they develop in cells already carrying a primary abnormality. In later disease stages, however, they may be so numerous as to completely dominate the karyotypic picture. Although less specific than primary changes, secondary aberrations nevertheless demonstrate nonrandom features with distribution patterns that appear to be dependent both on which primary abnormality is present and on the type of neoplasm”.

The report of a new primary chromosomal aberration in a given cancer is of scientific as well as clinical interest. The aberration may give rise to a novel fusion protein, alternatively abrogation of an otherwise normal gene product, and thus define a new genetic subgroup in such malignancies [2]. An example is the cryptic t(7;21)(p22;q22)/*RUNX1*-*USP42* chromosomal translocation which was first described in an AML patient and currently is considered a rare but nonrandom feature of myeloid malignancies where it is frequently found together with del(5q) [3–7].

We here present the molecular genetic and clinical features of a case of myelodysplastic syndrome with a novel primary t(7;13)(p14;q12) chromosome translocation that recombined the proteasome subunit alpha 2 (*PSMA2*) gene on 7p14 and the PAN3 poly(A) specific ribonuclease subunit (*PAN3*) gene on 13q12 generating a *PAN3*–*PSMA2* fusion gene. The disease later evolved into AML at which point also secondary chromosome abnormalities could be seen.

## Case presentation

A 74-year-old female patient was in April 2016 referred to our hospital because of thrombocytopenia. Blood analysis showed hemoglobin 10.8 g/dL, thrombocytes 31 × 10^9^/L, and white blood cells 3.8 × 10^9^/L with a normal differential count. Examination of a bone marrow biopsy showed 16% CD34-positive cells and dysplasia affecting mainly the megakaryocytic lineage, and the patient was diagnosed with myelodysplastic syndrome with excess of blasts 2 (MDS-EB 2). Cytogenetic analysis at this time showed the karyotype 46,XX,t(7;13)(p14;q12) (see below). No treatment was given.

One month later, examination of a new bone marrow aspirate showed 55% blasts and she was diagnosed with AML. Treatment with 5-azacytidine 5 days every 4th week was begun. However, a bone marrow aspirate after 6 monthly courses of 5-azacytidine showed 70% blasts, and because of disease progression she was now changed to intensive chemotherapy with age adjusted “3+7” (daunorubicin 50 mg/m^2^ day 1–3 and cytarabine 200 mg/m^2^ day 1–7), followed by one course of consolidation chemotherapy with mitoxantrone 10 mg/m^2^ day 1–5 and etoposide 100 mg/m^2^ day 1–5. In April 2017, 2 months after consolidation therapy, a new bone marrow aspirate again showed more than 50% blasts indicating AML recurrence. She was therefore started on decitabine 20 mg/m^2^ for 5 days every 4th week and after four courses, a new bone marrow biopsy showed 3–5% CD34+ cells. After another 2 months, a bone marrow biopsy still showed 5% CD34+ cells. Decitabine is continued and the patient is at the time of writing enjoying an active life in remission from her leukemia.

Bone marrow cells were cytogenetically investigated by standard methods [8, 9] and karyotyped according to the International System for Human Cytogenomic Nomenclature guidelines [10].

As part of our standard cytogenetic diagnosis of cases where AML is suspected, initial interphase FISH analyses of bone marrow cells were performed using the Cytocell multiprobe AML/MDS panel (Cytocell, http://www.cytocell.com) looking for -5/del(5q), -7/del(7q), del(20q), deletion of 17p13 (*TP53*), *MLL* rearrangements, *PML*-*RARA* fusion created by t(15;17)(q24;q11), *RUNX1*-*RUNX1T1* created by t(8;21)(q22;q22), and *CBFB*-*MYH11* generated by the inversion inv(16)(p13q22).

Further FISH analyses were performed using *PAN3* and *PSMA2* home-made break apart/double fusion probes. The BAC clones were purchased from BACPAC Resources Center (https://bacpacresources.org/home.htm). For the *PAN3* gene on chromosome 13, the BAC clones were RP11-179F17 (accession number AL356915, position: chr13:28039868–28234287; band: 13q12.2; GRCh38/hg38 Assembly) and RP11-502P18 (accession number AL138712, position: chr13:28234288–28396806; band: 13q12.2–13q12.3; GRCh38/hg38 Assembly); they were labelled green. For the *PSMA2* gene on chromosome 7, the BAC clones were RP11-111K18 (accession number ac010132, position: chr7:42801009–42967488; band: 7p14.1) and RP11-1081H3 (position: chr7:42923006–43086522; band: 7p14.1); they were labelled red. BAC DNA was extracted and probes were labelled with Fluorescein-12-dCTP (PerkinElmer, Boston, MA, USA) and Texas Red-5-dCTP (PerkinElmer) in order to obtain green and red signals, respectively, using Abbott’s nick translation kit (Des Plaines, IL, USA) and hybridized according to the companyʼs recommendations (http://www.abbottmolecular.com/home.html). FISH mapping of the clones on normal controls was performed to confirm their chromosomal location. Fluorescent signals were captured and analyzed using the CytoVision system (Leica Biosystems, Newcastle, UK).

Total RNA was extracted from the patientʼs bone marrow at the time when MSD was diagnosed using miRNeasy Mini Kit (Qiagen Nordic, Oslo, Norway). The RNA quality was evaluated using the Agilent 2100 Bioanalyzer (Agilent Technologies, Santa Clara, CA, USA) and 1 µg of total RNA was sent to the Genomics Core Facility at the Norwegian Radium Hospital, Oslo University Hospital (http://genomics.no/oslo/) for high-throughput paired-end RNA-sequencing. The RNA sequencing data were analyzed using the FusionCatcher software in order to discover fusion transcripts [11, 12] (https://github.com/ndaniel/fusioncatcher).

The primers used for PCR amplification and sequencing are listed in Table 1. The procedures of reverse transcriptase-polymerase chain reaction (RT-PCR) and direct sequencing of the PCR products were previously described [13]. For amplification of the *PAN3*–*PSMA2* fusion transcript, the primer sets PAN3-957F1/PSMA2-206R1 and PAN3-1005F1/PSMA2-168R1 were used. For amplification of the putative reciprocal *PSMA2*–*PAN3* fusion transcript, the primer set PSMA2-21F1/PAN3-1241R1 was used. For amplification of the *PAN3* and *PSMA2* transcripts, the primer sets PAN3-957F1/PAN3-1241R1 and PSMA2-21F1/PSMA2-206R1 were used, respectively. The PCR cycling involved initial denaturation at 94 °C for 30 s, followed by 35 cycles of 7 s at 98 °C, 30 s at 55 °C, 30 s at 72 °C, and a final extension for 5 min at 72 °C.

**Table 1:** Primers used for PCR amplification and Sanger sequencing analyses

Name	Sequence (5′ → 3′)	Position	Reference sequence	Gene
PAN3-957F1	CCCATCAATATGGTTTGGTGGA	957–978	NM_175854.7	*PAN3*
PAN3-1005F1	ACACCAAATCCTACTGCAAGCG	1005–1026	NM_175854.7	*PAN3*
PAN3-1241R1	CCATGTAACTTGCTGGATTTGGA	1263–1241	NM_175854.7	*PAN3*
PSMA2-206R1	CTTCGCTCATCATACAGAATGGATT	230–206	NM_002787.4	*PSMA2*
PSMA2-168R1	GTTGCTAATACCACACCATTTGCA	168–191	NM_002787.4	*PSMA2*
PSMA2-21F1	GTGCTTTGGCTCTTCGGGTAA	26–46	NM_002787.4	*PSMA2*

**Fig. 1:**
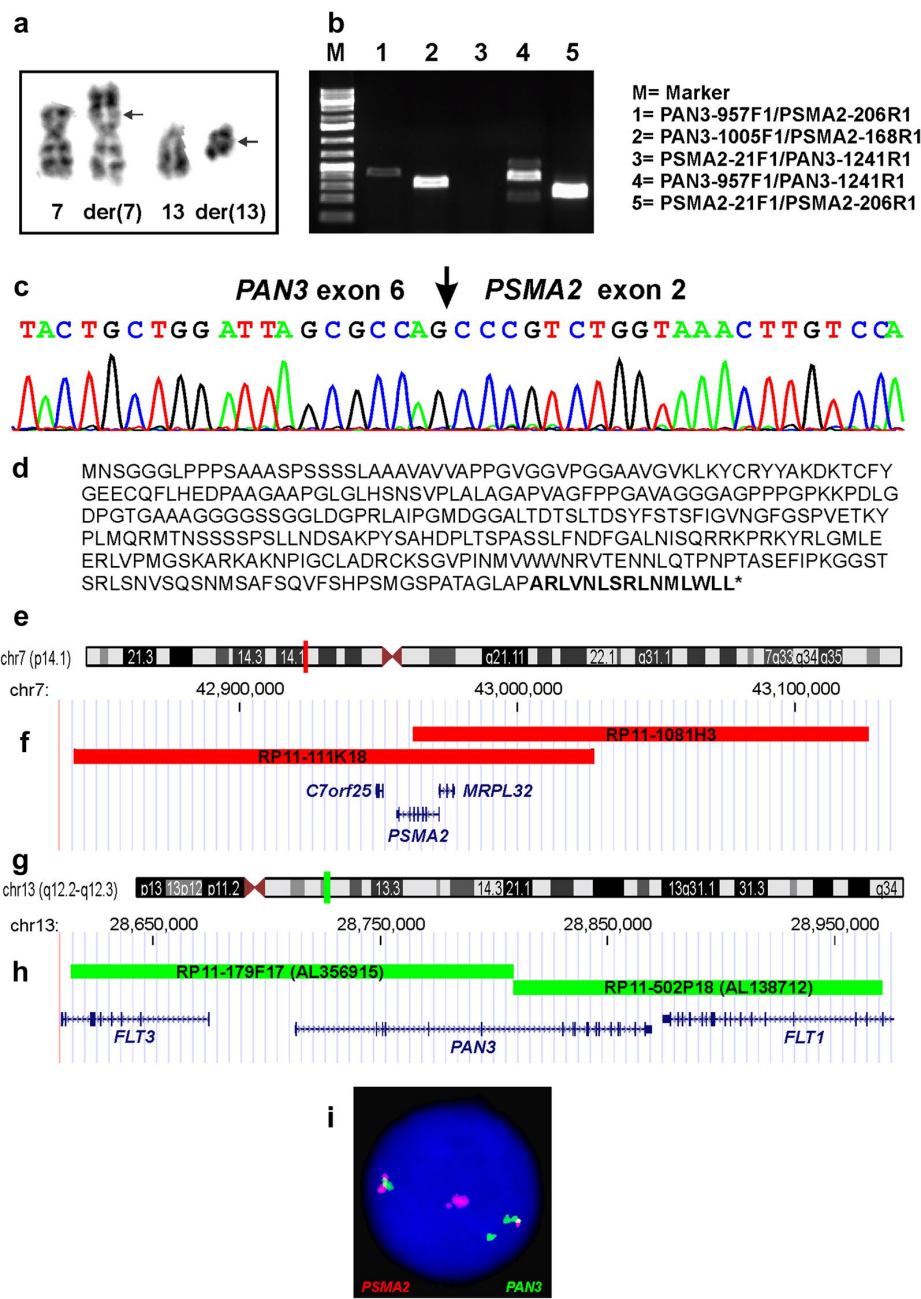
G-banding, molecular, and FISH analyses. **a** Partial karyotype showing the der(7) t(7;13)(p14;q12) and der(13)t(7;13)(p14;q12) together with the corresponding normal chromosome homologs. Breakpoint positions are indicated by arrows. **b** Gel electrophoresis showing the amplified cDNA fragments. **c** Partial sequence chromatogram of the amplified cDNA fragment showing the junction point of exon 6 of the *PAN3* gene with exon 2 of *PSMA2*. **d** Deduced amino acid sequence of the *PAN3*–*PSMA2* fusion transcript. The out-of-frame sequence from PSMA2 is in bold. The asterisk indicates the TAG termination codon. **e** Ideogram of chromosome 7 showing the mapping position of the *PSMA2* gene (vertical red line). **f** Diagram showing the FISH probe for *PSMA2*. Additional genes in this region are also shown. **g** Ideogram of chromosome 13 showing the mapping position of the *PAN3* gene (vertical green line). **h** Diagram showing the FISH probe for *PAN3*. Additional genes in this region are also shown. **i** FISH on interphase nucleus showing a red *PSMA2* signal, a green *PAN3* signal, and two yellow fusion signals

G-banding analysis of the bone marrow cells at diagnosis of MDS-EB2 yielded a karyotype with a single clonal abnormality: 46,XX,t(7;13)(p14;q12)[2]/46,XX[23] (Fig. 1a). G-banding analysis of a short-term cultured bone marrow aspirate drawn 4 months afterwards showed two cytogenetically related clones. In the first, two cells carried the chromosomal translocation t(7;13)(p14;q12) together with a der(13)ins(13;?)(q12;?). In the second subclone, ten cells carried a dic(19;20)(p13;q13) together with the two above-mentioned aberrations. The resulting karyotype was 46,XX,t(7;13)(p14;q12),der(13)ins(13;?)(q12;?)[2]/45,idem,dic(19;20)(p13;q13)[10]. G-banding analysis of a new bone marrow sample 9 months after the diagnosis yielded the karyotype 45,XX,t(7;13)(p14;q12),der(13)ins(13;?)(q12;?),dic(19;20)(p13;q13)[2]/46,XX[8]. Thus, G-banding analysis of bone marrow cells aspirated on several occasions indicated that t(7;13)(p14;q12) was the primary cytogenetic abnormality.

FISH analysis with multiprobes of the AML/MDS panel did not show -5/del(5q), -7/del(7q), del(20q), deletion of *TP53*, *MLL* rearrangements, or *PML*-*RARA*, *RUNX1*-*RUNX1T1* or *CBFB*-*MYH11* fusion (data not shown).

Using the FusionCatcher software on the fastq files of RNA sequencing data, 24 fusion genes were found (data not shown), among them fusion of *PAN3* from chromosome band 13q12 with the *PSMA2* gene from 7p14.

PCR with the primer combinations PAN3-957F1/PSMA2-206R1 and PAN3-1005F1/PSMA2-168R1 amplified 337 and 250 bp long cDNA fragments, respectively (Fig. 1b). Direct sequencing of the PCR products verified the presence of *PAN3*–*PSMA2* (Fig. 1c). The fusion point was identical to that found by analysis of RNA sequencing data using FusionCatcher software (Fig. 1c). In the *PAN3*–*PSMA2* transcript, exon 6 of *PAN3* (nt 1152 in sequence with accession number NM_175854 version 7) was fused out-of-frame to exon 2 of *PSMA2* (nt 90 in NM_002787 version 4) (Fig. 1c, d).

No cDNA amplified product was obtained when *PSMA2* forward and *PAN3* reverse primers (primer set PSMA2-21F1/PAN3-1241R1) were used, suggesting that the *PSMA2*–*PAN3* fusion transcript was absent or not expressed (Fig. 1b). Both normal *PAN3* (primer set PAN3-957F1/PAN3-1241R1) and *PSMA2* (primer set PSMA2-21F1/PSMA2-206R1) cDNA fragments were amplified from the cDNAs of the patient suggesting that both genes are expressed in the bone marrow (Fig. 1b).

Using break apart/double fusion homemade probes for the *PAN3* and *PSMA2* genes (Fig. 1e–i), interphase FISH analysis was performed on cells from the second bone marrow sample with the karyotype 46,XX,t(7;13)(p14;q12),der(13)ins(13;?)(q12;?)[2]/45,idem,dic(19;20)(p13;q13)[10].

A green (*PAN3* probe), a red (*PSMA2* probe), and two yellow fusion signals were seen in 48 out of 68 examined nuclei (Fig. 1i).

## Discussion and conclusions

We present here a case of MDS-EB2 progressing to AML in which a novel t(7;13)(p14;q12) was the primary acquired chromosomal change. At diagnosis of MDS, t(7;13) was the sole anomaly whereas 4 months later, when the patient had AML, the leukemic cells had acquired in their karyotype, in addition to t(7;13), the secondary changes der(13)ins(13;?)(q12;?) and dic(19;20)(p13;q13). RNA sequencing, RT-PCR, and FISH analyses showed that the t(7;13) rearranged the proteasome subunit alpha 2 (*PSMA2*) gene on 7p14 and the PAN3 poly(A) specific ribonuclease subunit (*PAN3*) gene on 13q12 to generate a *PAN3*–*PSMA2* fusion gene.

*PAN3* codes for the regulatory subunit of the poly(A)-nuclease (PAN) deadenylation complex (PAN2–PAN3 complex), one of two cytoplasmic eukaryotic poly(A) nuclease complexes involved in mRNA decay [14]. The PAN2–PAN3 complex specifically shortens poly(A) tails of RNA when the poly(A) stretch is bound by poly(A)-binding protein (PABP), followed by rapid degradation of the shortened mRNA tails by the CCR4-NOT complex [15, 16]. The N‐terminus of PAN3 contains a zinc finger and a PABP interacting motif 2 (PAM2). The C‐terminal part contains a pseudokinase, a coiled coil, and a C‐terminal knob domain [16–21]. The PAN3 pseudokinase domain binds ATP, a function required for mRNA degradation in vivo [16–21]. However, it does not have kinase activity due to structural rearrangements and loss of active site residues [16–21]. The coiled coil mediates dimerization and the C-terminal knob domain binds to the WD40 domain of PAN2 [16–21]. PAN3 acts as a positive regulator of PAN activity, recruiting the catalytic subunit PAN2 to mRNA via its interaction with PABP and to miRNA targets via its interaction with GW182 family proteins [16–21]. Heterozygous deletions of the *PAN3* gene were reported in 5.4% of cases of highly hyperdiploid childhood acute lymphoblastic leukemia. Real-time quantitative RT-PCR analyses showed that in cases with deletion of *PAN3*, the gene was expressed at a lower level than in the samples without such deletion [22].

*PSMA2* is ubiquitously expressed and encodes a peptidase which is a component of the alpha subunit of the 20S core proteasome complex [23]. The proteasome is a multi-catalytic proteinase complex. It is distributed throughout eukaryotic cells at a high concentration and cleaves peptides in an ATP/ubiquitin-dependent process as part of a non-lysosomal pathway [24, 25]. It plays a central role in the control of numerous cellular activities including regulation of the cell cycle [24, 25]. Inhibition of proteasome was found to be an effective therapeutic strategy in many hematologic malignancies [26–29].

The *PAN3*–*PSMA2* fusion transcript codes for a putative PAN3 truncated protein which contains amino acid residues 1–333 from PAN3 protein (accession number NP_787050 version 6) corresponding to exons 1–3 of the gene, and 15 novel amino acid residues (ARLVNLSRLNMLWLL) stemming from the out-of-frame fusion of *PSMA2*. This putative protein would therefore contain the zinc finger and a PABP interacting motif 2 (PAM2) but would lack the normal C‐terminal part which contains the pseudokinase, the coiled coil, and the C‐terminal knob domain of PAN3. The precise role of the truncated PAN3 protein in the development of myelodysplasia/leukemia cannot be predicted without functional studies. However, an anomaly in deadenylation, which is fundamental to the regulation of gene expression, can be assumed. Alternatively, loss of a functional PAN3 and/or PSMA2 allele might be the important factor in pathogenesis. Whether any functional similarity exists between the present translocation and abrogation case and ALLs with deletion of an entire *PAN3* allele [22], is a moot point.

Chromosomal rearrangements resulting in gene truncation have been described repeatedly for the *RUNX1* and *ETV6* genes [30, 31]. The aberrations generate a premature stop codon in the open reading frames leading to expression of C-terminal truncated forms of the RUNX1 or ETV6 proteins [30, 32]. Truncated RUNX1 proteins were shown to interfere with normal RUNX1 [33–35]. Truncated forms of ETV6 were found to have a dominant-negative effect on normal ETV6 function and disrupt both primitive and definitive hematopoiesis in the zebrafish model [36]. Chromosome translocations resulting in gene truncation have also been reported for other genes. For example, a t(3;21)(q22;q22) leading to truncation of *RYK* was seen in atypical chronic myeloid leukemia [37]. In a case of AML transformed from myelodysplastic syndrome, a t(2;7)(p24.3;p14.2) generated an out-of-frame *NBAS*-*ELMO1* fusion transcript coding for a truncated NBAS protein [38]. Recently, in a case of AML, a t(3;5)(p24;q14) translocation was found to result in fusion of *SATB1* with an expression sequence tag. The *SATB1*-fusion transcript would code for a SATB1 protein lacking the C-terminal DNA-binding homeodomain [39].

The patient in this report did not respond to treatment with 5-azacitidine, daunorubicin plus cytarabine, or mitoxantrone plus etoposide. She did, however, have a very favorable response to decitabine. It is possible that the 7;13-translocation is causatively involved in this difference, but in the absence of other cases with the same genetic change, one cannot tell. The case anyway illustrates that some patients with MDS or AML who do not respond favorably to standard treatment, may benefit from a change to decitabine [40, 41]. It may be particularly noteworthy from a clinical point of view that some patients who do not respond to 5-azacitidine, may do so to decitabine in spite of the fact that both drugs are hypomethylating agents.

## Abbreviations

ALL: acute lymphoblastic leukemia
AML: acute myeloid leukemia
BAC: bacterial artificial chromosome
FISH: fluorescence in situ hybridization
MDS: myelodysplastic syndrome
RT-PCR: reverse transcriptase-polymerase chain reaction
